# A Novel Role of Growth Differentiation Factor (GDF)-15 in Overlap with Sedentary Lifestyle and Cognitive Risk in COPD

**DOI:** 10.3390/jcm9092737

**Published:** 2020-08-24

**Authors:** Tsunahiko Hirano, Keiko Doi, Kazuto Matsunaga, Shun Takahashi, Tomohiro Donishi, Kazuyoshi Suga, Keiji Oishi, Kasumi Yasuda, Yusuke Mimura, Misa Harada, Sumiteru Suizu, Keita Murakawa, Ayumi Chikumoto, Yuichi Ohteru, Kazuki Matsuda, Sho Uehara, Kazuki Hamada, Shuichiro Ohata, Yoriyuki Murata, Yoshikazu Yamaji, Maki Asami-Noyama, Nobutaka Edakuni, Tomoyuki Kakugawa

**Affiliations:** 1 Department of Respiratory Medicine and Infectious Disease, Graduate School of Medicine, Yamaguchi University, Ube 755-8505, Japan; decem119@yamaguchi-u.ac.jp (K.D.); kazmatsu@yamaguchi-u.ac.jp (K.M.); hara-da@yamaguchi-u.ac.jp (M.H.); relativity.theory135@gmail.com (S.S.); murakawakeita124@gmail.com (K.M.); chiku05@yamaguchi-u.ac.jp (A.C.); yohteru@yamaguchi-u.ac.jp (Y.O.); k0m1a2t8s1u1d2a1@gmail.com (K.M.); n010eb.mie@gmail.com (S.U.); khamada@yamaguchi-u.ac.jp (K.H.); j015ebponyou@gmail.com (S.O.); yyamaji@yamaguchi-u.ac.jp (Y.Y.); noyamama@yamaguchi-u.ac.jp (M.A.-N.); edakuni@yamaguchi-u.ac.jp (N.E.); 2 Department of Neuropsychiatry, Wakayama Medical University, Wakayama 641-8510, Japan; t-shun@wakayama-med.ac.jp (S.T.); y-kasumi@wakayama-med.ac.jp (K.Y.); 3 Department of System Neurophysiology, Graduate School of Wakayama Medical University, Wakayama 641-8510, Japan; tdonishi@wakayama-med.ac.jp; 4 Department of Radiology, Semui PET Screening and Radiatiotherapeutic Site, St. Hill Hospital, Ube 755-0155, Japan; sugar@sthill-hp.or.jp; 5 Department of Medicine and Clinical Science, Graduate School of Medicine, Yamaguchi University, Ube 755-8505, Japan; ohishk@yamaguchi-u.ac.jp (K.O.); ymurata-ygc@umin.ac.jp (Y.M.); 6 Department of Clinical Research, National Hospital Organization Yamaguchi Ube Medical Center, Ube 755-0241, Japan; mimuray-yumc@umin.ac.jp; 7 Department of Pulmonology and Gerontology Graduate School of Medicine, Yamaguchi University, Ube 755-8505, Japan; tomoyukikakugawa@gmail.com

**Keywords:** GDF-15, sedentary, cognitive impairment, COPD, comorbidity, motoric cognitive risk, aging

## Abstract

Sedentary behavior and cognitive impairment have a direct impact on patients’ outcomes. An energy metabolic disorder may be involved in the overlap of these comorbid conditions (motoric cognitive risk (MCR)) in patients with chronic obstructive pulmonary disease (COPD). We aimed to explore the linkage between a proapoptotic protein, growth differentiation factor (GDF)-15, and MCR. Physical activity (PA), cognitive function (Japanese version of the Montreal Cognitive Assessment: MOCA-J), and the serum GDF-15 levels were assessed in healthy subjects (*n* = 14), asthmatics (*n* = 22), and COPD patients (*n* = 28). In the entire cohort, serum GDF-15 had negative correlations with exercise (Ex) (*ρ* = −0.43, *p* < 0.001) and MoCA-J (*ρ* = −0.44, *p* < 0.001), and Ex and MOCA-J showed a positive correlation (*ρ* = 0.52, *p* < 0.0001). Compared to healthy subjects and asthmatics, COPD patients showed the highest serum GDF-15 levels and had a significantly higher proportion of subjects with MCR (both sedentary lifestyle (EX < 1.5) and cognitive risk (MoCA-J ≤ 25)). Also, we found that serum GDF-15 has a screening potential (100% sensitivity) greater than aging (67% sensitivity) for detecting MCR in COPD patients. In conclusion, higher serum GDF-15 had interrelationships with a sedentary lifestyle and cognitive risk. This protein was not disease-specific but could be a screening biomarker to detect MCR related to poor health outcomes of COPD patients.

## 1. Introduction

Chronic obstructive pulmonary disease (COPD) is a major cause of death and loss of social activities [1,2,3]. In fact, it is well known that representative comorbid conditions, sedentary behavior and cognitive impairment, have a direct impact on patients’ health outcomes [4,5,6,7]. Importantly, these two comorbid conditions commonly co-occur in the same patient, and their overlap is now recognized as motoric cognitive risk (MCR) syndrome [8]. Handschin et al. reported that a sedentary lifestyle may be a trigger for the progression of chronic disease through systemic inflammation accompanied by skeletal muscle inactivity [9]. Physical inactivity may be associated with MCR via molecular biological responses between the brain and skeletal muscle.

Growth differentiation factor-15 (GDF-15), which is a member of the transforming growth factor-β superfamily [10], is suggested to be a promising biomarker for the prognosis [11]. This protein, expressed in multiple tissues including brain, lung, and muscle [12,13,14], is induced during tissue injury, inflammation, and oxidative stress [15]. Previous reports have shown that GDF-15 is also associated with cell apoptosis, senescence, and energy metabolic disorder due to mitochondrial dysfunction, which are hallmarks of aging [13,16,17,18,19]. Actually, GDF-15 has been shown to be a promising biomarker of mortality in aging-associated disease [20]. If systemic inflammation and energy metabolic disorder due to muscle inactivity are involved in the mechanism of MCR, this proapoptotic protein may be a useful biomarker to predict the risk of poor health outcomes in COPD patients.

This study aimed to explore the linkage of GDF-15 with a sedentary lifestyle and cognitive risk, and to verify the diagnostic ability of GDF-15 to detect MCR in COPD patients.

## 2. Materials and Methods

### 2.1. Study Subjects

Subjects older than 40 years were recruited from Yamaguchi Medical University Hospital. The study protocol and its amendments were approved by the local ethics committee at Yamaguchi Medical University (institutional review board no. H28-031). The study was registered in the UMIN Clinical Trials Registry: UMIN000024645.

The subjects consisted of healthy subjects, asthmatics and patients with COPD. Asthma and COPD were diagnosed by a pulmonologist. Asthmatic patients had documented reversible airflow limitation and were treated based on the Global Initiative for Asthma (GINA) guidelines [21]. Asthmatics had adequate inhalation and good adherence to asthma therapy. The COPD patients were treated based on the Global Initiative for Chronic Obstructive Lung Disease (GOLD) guidelines [22]. Asthmatics and patients with COPD were stable and had no exacerbations for at least three months prior to the study. Patients with other pulmonary diseases such as interstitial lung disease or with disorders that would prevent them from completing the study assessments were excluded. Patients received an explanation of the study protocol and gave written informed consent.

### 2.2. Evaluation of Physical Activity

The Active Style Pro HJA-750C^®^ with triaxial acceleration can be worn around the waist and continually record physical activity for two weeks. We measured the value of the metabolic equivalents (METs) and calculated the exercise (EX) that was the value of metabolic equivalents multiplied by their durations (MET × hours per day) as previously reported [23,24,25,26,27,28] According to the American College of Sports Medicine and the American Heart Association recommendation [29], we defined EX < 1.5 as a sedentary lifestyle.

### 2.3. Assessment of Ccognitive Function

Trained research assistants administered the Japanese version of the Montreal Cognitive Assessment (MoCA-J) according to standardized procedures for cognitive function. MoCA-J assesses short-term memory, visuospatial ability, some executive functions, attention, concentration, working memory, language, and temporal and spatial orientation. Since a MoCA-J score with a threshold is less than 25 is considered to indicate mild cognitive impairment [30,31], we defined a MoCA-J score ≤ 25 as cognitive risk.

### 2.4. Definition of MCR

We defined cases that fulfilled both EX < 1.5 (sedentary lifestyle) and a MoCA-J score ≤ 25 (cognitive risk) at the same time as MCR in this study.

### 2.5. Measurement of GDF-15

Peripheral venous blood was drawn into pyrogen-free EDTA collection tubes and centrifuged within 30 min at 2150× *g* for 15 min at 4 °C. The plasma was aliquoted and stored in −80 °C ultra-freezers. The plasma levels of GDF-15 were measured in the baseline samples by ELISA (Quantikine, R&D Systems, Inc., Abingdon, UK). All samples were measured in duplicate and accepted only if the intra-assay variance was <10%.

### 2.6. Assessment of Pulmonary Function

Under the recommendations of the American Thoracic Society/European Respiratory Society, the pulmonary function was assessed by CHESTAC-8800 DN type (Chest Ltd., Tokyo, Japan) [32].

### 2.7. Statistical Analysis

Data are shown as the median ± interquartile (IQR). We divided the subjects into disease groups or groups based on the EX or MoCA-J score. The Wilcoxon’s rank sum test or Kruskal–Wallis test was employed to measure the differences in each group. The Kruskal–Wallis test with post-hoc analysis was used to compare the differences between specific groups. Spearman’s rank correlation and multiple linear regression analysis using the least square method were performed to analyze the correlation between parameters. We analyzed sensitivity versus specificity by the area under the curve (AUC) and found a cutoff value for GDF-15 that could detect MCR. Statistics were performed using JMP Pro^®^, version 14.0.0 (SAS Institute, Inc., Cary, NC, US). A probability value less than 0.05 was considered statistically significant.

## 3. Results

The baseline characteristics are shown in Table 1. A total of 64 subjects were enrolled in the study. The subjects consisted of 14 healthy subjects, 22 asthmatics, and 28 patients with COPD. The subjects were 70.3% male and had a mean age of 67.4 years. The median of body mass index (BMI) was 23.1 and a total of 41(64%) subjects had a smoking history.

Table 2 shows the results of physical activity (EX), cognition level (MoCA-J), and GDF-15 among subjects. The medians of EX, MoCA-J, and GDF-15 were 3.2 (MET × hours per day), 25 (scores), and 934 (pg/mL), respectively, in all subjects. The GDF-15 intra-assay variance of all samples was <10%. EX (2.0 METs × hours per day) and MoCA-J (23 scores) were lowest in COPD among the subjects (*p* < 0.0005, *p* < 0.0001, respectively). Comparing subjects by the GDF-15 levels, those in COPD (1285 pg/mL) showed trend of the highest values among the subjects (*p* < 0.05) and GDF-15 levels in COPD were higher than those in healthy subjects and asthmatics by post-hoc analysis of differences between the specific groups (830 pg/mL; *p* < 0.005, 793 pg/mL; *p* = 0.08, respectively). 

In the entire cohort, the associations of GDF-15, EX, and MoCA-J were strong, although there was no correlation between these relationships after adjustment for age (Table 3).

**Figure 1:**
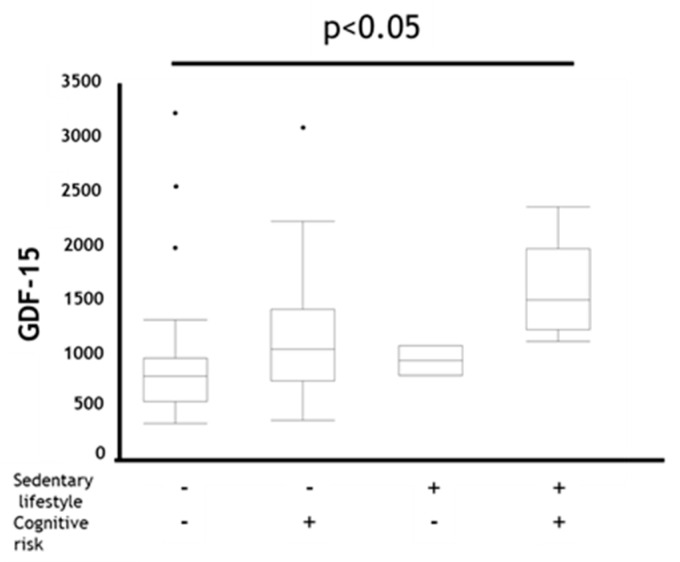
Comparison of GDF-15 in coexistence status with sedentary lifestyle and cognitive risk in all subjects. Data are presented as box plot including median (IQR). *P*-values compared among subjects by Kruskal–Wallis test. GDF-15: Growth Differentiation Factor-15, Sedentary lifestyle + : EX (MET × hours per day) < 1.5, Sedentary lifestyle − : EX (MET × hours per day) ≥ 1.5, Cognitive risk + : MoCA-J scores ≤ 25, Cognitive risk − : MoCA-J scores > 25.

When comparing the difference in GDF-15 between categories based on physical activity or cognitive risk level, participants who belonged to the MCR category showed trend of the highest GDF-15 levels (Figure 1). Therefore, we assessed the diagnostic ability of GDF-15 and age to determine whether MCR existed or not using receiver operating characteristic (ROC) analysis. As a result, although both AUCs were almost the same, the sensitivity of GDF-15 was better than that of age (Table 4).

**Figure 2:**
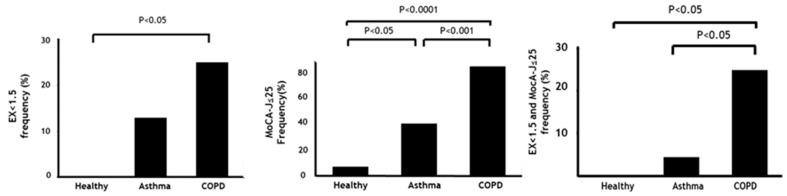
Frequency of sedentary lifestyle, cognitive risk, and MCR among subjects. Data are presented as frequency. P-values show post-hoc analysis between each group. MET: Metabolic equivalent, EX: Exercise (MET × hours), MoCA-J: Japanese version of the Montreal Cognitive Assessment, MCR: Motoric cognitive risk syndrome.

As shown in Figure 2, the frequency of sedentary lifestyle (EX < 1.5) (25%) or cognitive risk (MoCA-J ≤ 25) (86%) was highest in COPD among the groups (healthy, 0%; 7% asthma, 14%; 41%, respectively). Importantly, MCR (25%) was seen most frequently in COPD compared to the healthy subjects and asthmatics (0%, 4.6%, respectively).

Significant associations of GDF-15, EX and MoCA-J were found in COPD patients, but similar to in the result of the entire cohort, age correction weakened these relationships (Table 5).

**Figure 3:**
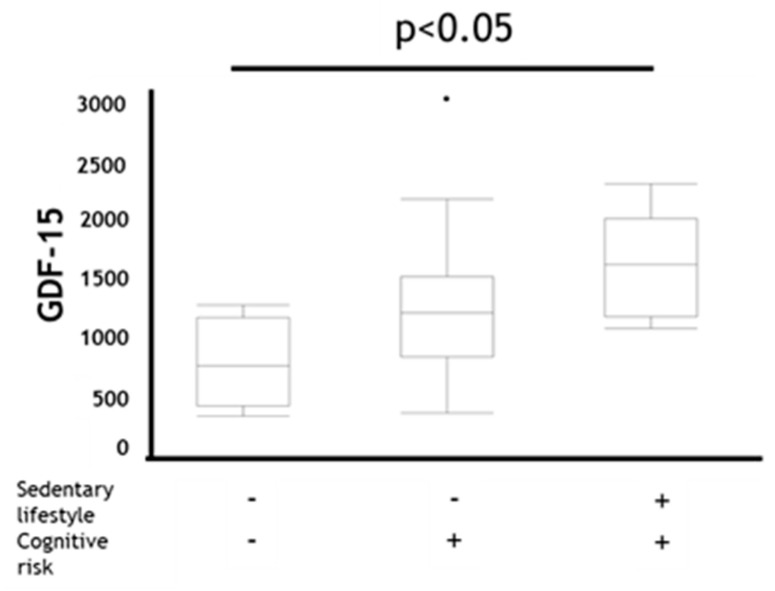
Comparison of GDF-15 in coexistence status with sedentary lifestyle and cognitive risk in COPD. Data are presented as boxplot including median (IQR). *P*-values compared among subjects by Kruskal–Wallis test. GDF-15: Growth Differentiation Factor-15 Sedentary lifestyle +: EX (MET × hours per day) < 1.5, Sedentary lifestyle − : EX (METs × hours per day) ≥ 1.5, Cognitive risk + : MoCA-J scores ≤ 25, Cognitive risk −: MoCA-J scores > 25.

Additionally, trends of the GDF-15 levels in the MCR category were significantly more likely to be higher than those in other categories in patients with COPD (*p* < 0.05) (Figure 3).

**Figure 4:**
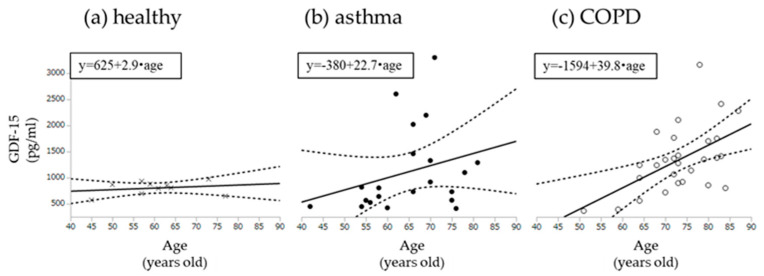
Univariable models for age with GDF-15. (**a**) Correlation with age and GDF-15 among healthy subjects; (**b**) Correlation with age and GDF-15 among asthma; (**c**) Correlation with age and GDF-15 among COPD. GDF-15: Growth Differentiation Factor-15. Dotted lines show 95% confidence intervals.

**Figure 5:**
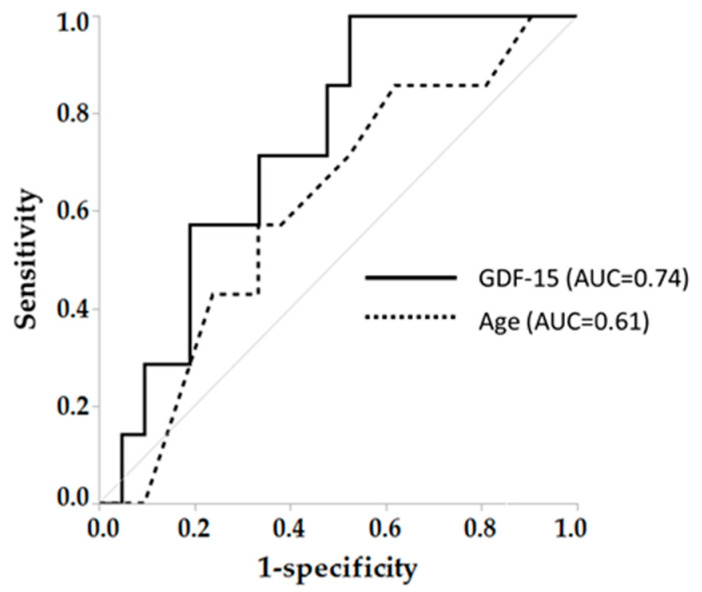
Diagnostic ability in GDF-15 and age to identify MCR in COPD. AUC: Area under the curve, MCR: Motoric cognitive risk syndrome. Arrows mean cut-off point to identify coexistence of sedentary lifestyle and cognitive risk (MCR).

In order to clarify the role of age on the results, we performed univariate and multivariate analysis of age with MCR related parameters including EX, MoCA-J and GDF-15. As a result, in both all subjects and COPD, age was independently associated with GDF-15 (F = 5.6, *p* < 0.05, F = 4.9, *p* < 0.05, respectively) (Table S1). However, when comparing the relationship between age and GDF-15 for each subject group, the slope of GDF-15-Age (39.8) in COPD group was steeper than those in other groups (healthy; 2.9, asthma; 22.7, respectively) (Figure 4). Finally, a GDF-15 level > 1118 pg/mL secured 100% sensitivity and 50% specificity to detect MCR in COPD (AUC = 0.74), while AUC of age was 0.61 (57% sensitivity, 67% specificity) (Figure 5).

## 4. Discussion

### 4.1. Principal Findings

We observed that higher serum GDF-15 levels showed interrelationships with a sedentary lifestyle and cognitive risk. These data suggest that a systemic effect and an energy metabolic disorder may be involved in a pathway associated with these comorbid conditions. Compared to healthy subjects and asthmatics, the COPD patients showed the highest serum GDF-15 levels, which tended to be steeper with aging and had a significantly higher proportion of subjects with MCR. We found that this proapoptotic protein was not disease-specific but appeared to have the superior ability to detect MCR than aging in COPD patients.

### 4.2. Comparison with Other Studies

The mechanisms underlying the effects of GDF-15 secretion on neuron, muscle, and airway are not fully understood. The secretary mechanism of GDF-15 may be associated with skeletal muscle dysfunction by physical inactivity, because the molecule is a footprint of an energy metabolic disorder due to mitochondrial dysfunction [9]. On the other hand, Mullican et al. recently identified glial cell line-derived neurotrophic factor family receptor-a–like (GFRAL), the receptor tyrosine kinase RET, as a specific receptor in the brain for GDF-15 [33]. They described that the GDF-15/GFRAL pathway regulates appetite and could be involved with energy metabolic disorder. These data may help to explain our findings that higher serum GDF-15 levels showed interrelationships and with a sedentary lifestyle and cognitive risk.

The detailed biological mechanisms by which the GDF-15 pathway links with the coexistence of physical inactivity and cognitive risk in COPD remain unclear. However, it is suggested that there is a pathophysiological basis for the comorbidity through systemic inflammation accompanied by physical inactivity in chronic disease including COPD [9,34,35]. In addition, mobility and cognition tasks share common brain regions, such as the cortex in order to execute each function [36], and this area may be susceptible to systemic inflammation [37,38]. Actually, systemic inflammation is suggested to have a pathogenic role in accelerating pathologic processes underlying cognitive decline [35]. Mitochondrial dysfunction is also reported to contribute to neural injury via neuronal apoptosis [39,40,41], which may be closely linked with the biological responses between muscle and neuron. Therefore, our findings could support the hypothesis that there is inter-organ crosstalk between the brain and pulmonary and skeletal muscle [9,34,35].

### 4.3. Strength and Limitations

To the best of our knowledge, this is the first study that investigated the relationship between MCR and GDF-15 in COPD. An overlap of these comorbid conditions may have greater predictive value for worsening QOL and prognosis than a single comorbidity in patients with COPD because multiple treatments may be necessary to deal with various diseases, which could complicate the management of such patients [42]. In addition, such conditions are more likely to share pathways of accelerated aging [43]. Therefore, in this study, we expanded the evidence that GDF-15 could be a novel biomarker for detecting MCR and elucidating the mechanism of accelerated aging in COPD. The detection of MCR using GDF-15 may lead to novel precautions and interventions that target common pathways of accelerated aging.

Some previous reports have shown that elevated serum GDF-15 levels contribute to subclinical coronary atherosclerosis [44] and are correlated with adverse outcomes such as exacerbation and lung function decline in COPD [45]. Our results are in accord with these findings in that GDF-15 adds a novel measurable value to a predictor model based on a conventional method. Since a serum GDF-15 cutoff value to detect MCR showed high sensitivity, it is suggested that GDF-15 could be a useful screening tool for predementia in COPD.

The current study has some limitations. The first is the small number of subjects. Next, this cross-sectional study could not clarify a causal relationship between GDF-15 and MCR. More large-scaled longitudinal studies are needed. The GDF-15 was not a specific biomarker to identify MCR in the study subjects. In order to overcome this point, we need to explore whether GDF-15 combined with the results from brain imaging, for example, could be useful to specifically identify MCR. Finally, it is well known that aging is strongly associated with most outcomes in our study. In fact, when adjusted for age, the relationship between GDF-15 and comorbid conditions was greatly diminished. However, these data do not preclude the significance of GDF-15 as a screening biomarker to detect MCR. The sensitivity of GDF-15 to detect MCR was superior to that of age not only in entire cohort, but also in COPD. In addition, the AUC of GDF-15 in COPD was higher than that of age, and the increasing rate of GDF-15 with aging in COPD accelerated than that in other groups. This indicates that higher GDF-15 is originated from combination of aging and specific disease effect in COPD. Our study revealed the relationship between GDF-15 and comorbid conditions that are associated with heavy burdens on the lives of COPD patients.

## 5. Conclusions

Our study demonstrated that higher GDF-15 levels had interrelationships with a sedentary lifestyle and cognitive risk. We provide the novel insight that this protein is not disease-specific but could be a screening biomarker to detect MCR, which is related to poor health outcomes in COPD patients.

## Figures and Tables

**Table 1:** Characteristics of the study subjects.

Age (years)	69.5 (59–75)
Healthy/Asthma/COPD	14/22/28
Sex (M/F)	45/19
BMI (kg/m^2^)	23.1 (20.7–25.5)
Smoking status (Cu/Ex/Non)	10/31/23
Pack years	28.5 (0–44)
%VC (%)	101 (89–114)
%FVC (%)	100 (89–116)
%FEV_1_ (%)	89 (75–103)
%DLco/VA (%)	97 (69–109)

Data are presented as median (IQR, interquartile) unless otherwise stated. Definition of abbreviations: BMI: body mass index; Cu: Current smoker, Ex: Ex-smoker; Non: Nonsmoker; %: predicted; VC: vital capacity; FVC: forced vital capacity, FEV_1_ forced expiratory volume in 1 s; DLco: diffusing capacity of lung carbon monoxide; VA: alveolar volume.

**Table 2:** Comparison of physical activity (EX), cognition level (MoCA-J), and growth differentiation factor-15 (GDF-15) among subjects.

	All	Healthy	Asthma	COPD	*p*-Value
EX (MET × hours per day)	3.2 (1.9–4.9)	5.4 (3.5–7.5)	3.4 * (2.3–5.0)	2.0 ^§,||^ (1.4–3.3)	<0.0005
MoCA-J (scores)	25 (22–27)	26.5 (26–28.3)	26.5 (23.8–28)	23 ^‡^ (21–25)	<0.0001
GDF-15 (pg/mL)	934 (708–1384)	830 (684–893)	793 (541–1366)	1285^†^ (888–1700)	<0.05

Data are presented as median (IQR, interquartile). Differences between groups were assessed by Kruskal–Wallis test and Wilcoxon test with post-hoc analyses. *p*-values compared among subjects by Kruskal-Wallis test. * Significant difference of *p* < 0.05 as compared with healthy controls. † Significant difference of *p* < 0.005 as compared with healthy controls. ‡ Significant difference of *p* < 0.0001 as compared with healthy controls. ^§^ Significant difference of *p* < 0.0005 as compared with healthy controls. || Significant difference of *p* < 0.05 as compared with asthma. Significant difference of *p* < 0.005 as compared with asthma. *Definition of abbreviations:* EX: exercise; METs: metabolic equivalents; MoCA-J: Japanese version of the Montreal Cognitive Assessment; GDF-15: growth differentiation factor-15.

**Table 3:** Association of EX, MoCA-J, and GDF-15 unadjusted and adjusted by age in all subjects.

	Age Unadjusted		Age Adjusted	
	*ρ*	*p*-Value	F	*p*-Value
GDF-15 and EX	−0.43	<0.001	1.7	0.19
GDF-15 and MoCA-J	−0.44	<0.001	0.26	0.62
EX and MoCA-J	0.52	<0.0001	3.7	0.06

*ρ* means spearman’s rank correlation coefficient, F means F-statistic by least square method.

**Table 4:** Diagnostic ability in age and GDF-15 to identify motoric cognitive risk (MCR) in all subjects.

	AUC	Sensitivity	Specificity	Cut off Value
Age	0.80	88%	66%	72 (years)
GDF-15	0.79	100%	67%	1118 (pg/mL)

Definition of abbreviations: AUC; area under the curve.

**Table 5:** Association of EX, MoCA-J, and GDF-15 adjusted by age unadjusted and adjusted by age in chronic obstructive pulmonary disease (COPD).

	Age Unadjusted		Age Adjusted	
	*ρ*	*p*-Value	F	*P*-Value
GDF-15 and EX	−0.52	<0.01	1.87	0.18
GDF-15 and MoCA-J	−0.45	<0.05	0.67	0.42
EX and MoCA-J	0.43	<0.05	2.1	0.16

*ρ* means spearman’s rank correlation coefficient, F means F-statistic by least square method.

## Supplementary Materials

The following are available online at https://www.mdpi.com/2077-0383/9/9/2737/s1, Table S1. Association of age and EX, MoCA-J and GDF-15.
