# Supplementary material for: A Novel Role of Growth Differentiation Factor (GDF)-15 in Overlap with Sedentary Lifestyle and Cognitive Risk in COPD
Source: J Clin Med. 2020 Aug 24;9(9):2737. doi: 10.3390/jcm9092737 (PMC7565594; doi:10.3390/jcm9092737)
Supplement: Supplementary file 1 [file jcm-09-02737-s001.pdf]

**Supplemental Table S1**. Association of age and EX, MoCA-J and GDF-15

| | All subjects | | | | COPD | | | |
|---|---|---|---|---|---|---|---|---|
| | univariate | | multivariate | | univariate | | multivariate | |
| | $\varrho$ | P-value | F | P-value | $\varrho$ | P-value | F | P-value |
| EX | -0.49 | <0.0001 | 0.9 | n.s. | -0.40 | <0.05 | 0.0 | n.s. |
| MoCA-J | -0.69 | <0.0001 | 19.8 | <0.0001 | -0.60 | <0.001 | 4.1 | 0.05 |
| GDF-15 | 0.50 | <0.0001 | 5.6 | <0.05 | 0.47 | <0.05 | 4.9 | <0.05 |

$\varrho$ means spearman's rank correlation coefficient, F means F-statistic by least square method
